# Impact of normocapnic and permissive hypercapnic one-lung ventilation on arterial oxygenation

**DOI:** 10.1186/cc9595

**Published:** 2011-03-11

**Authors:** T Végh, Z Szabó-Maák, S Szatmári, J Hallay, I László, I Takács, B Fülesdi

**Affiliations:** 1 University of Debrecen, Hungary

## Introduction

Physiologically, an approximately 5 to 10 mmHg difference exists between end-tidal carbon dioxide (EtCO_2_) and arterial carbon dioxide (PaCO_2_) measured during double-lung ventilation (DLV) that may increase during one-lung ventilation (OLV) especially if low tidal volume is applied. There is no evidence that during OLV the EtCO_2 _or PaCO_2 _should be kept in the normal range. The aim of the present work was to test whether different ventilatory strategies to maintain EtCO_2 _or PaCO_2 _in the normal range during OLV have any impact on arterial oxygenation (PaO_2_).

## Methods

Data were obtained from 100 patients undergoing thoracic surgery necessitating OLV. Patients were randomized into two groups. In GrEtCO_2 _(*n *= 50) the OLV was guided by capnography, and the respiratory rate (RR) was adjusted to maintain EtCO_2 _in the normal range. In GrPaCO_2 _(*n *= 50) the OLV was guided by arterial blood gas analysis (ABG) and RR was adjusted to maintain PaCO_2 _in the normal range. ABG was performed in a supine position after induction and in a lateral decubitus position during DLV and every 15 minutes during OLV. During OLV 5 ml/kg tidal volume with 5 cmH_2_O PEEP, I:E = 1:2 ratio and FiO_2 _1.0 was used.

## Results

There were no significant differences in PaO_2 _values between groups during DLV and at the 15th minute of OLV. There were significant differences in PaO_2 _at the 30th and 45th minutes between groups. In GrPaCO_2 _mean airway pressure and RR was higher, and the inspiratory and expiratory time was shorter than in GrEtCO_2_.

## Conclusions

The relatively high RR impairs the emptying of alveoli and results in increased functional residual capacity. So the normocapnic lung-protective OLV results in significantly higher PaO_2 _than permissive hypercapnic OLV.
